# Anthropogenic Effects on Forest Ecosystems at Various Spatio-Temporal Scales

**DOI:** 10.1100/tsw.2002.129

**Published:** 2002-03-27

**Authors:** Michael Bredemeier

**Affiliations:** Forest Ecosystems Research Centre, University of Goettingen, Germany

**Keywords:** forest ecosystems, long-term, human impact, overexploitation, soil, acidification, nutrient loss, degradation, deforestation

## Abstract

The focus in this review of long-term effects on forest ecosystems is on human impact. As a classification of this differentiated and complex matter, three domains of long-term effects with different scales in space and time are distinguished: 1- Exploitation and conversion history of forests in areas of extended human settlement 2- Long-range air pollution and acid deposition in industrialized regions 3- Current global loss of forests and soil degradation.

There is an evident link between the first and the third point in the list. Cultivation of primary forestland — with its tremendous effects on land cover — took place in Europe many centuries ago and continued for centuries. Deforestation today is a phenomenon predominantly observed in the developing countries, yet it threatens biotic and soil resources on a global scale. Acidification of forest soils caused by long-range air pollution from anthropogenic emission sources is a regional to continental problem in industrialized parts of the world. As a result of emission reduction legislation, atmospheric acid deposition is currently on the retreat in the richer industrialized regions (e.g., Europe, U.S., Japan); however, because many other regions of the world are at present rapidly developing their polluting industries (e.g., China and India), “acid rain” will most probably remain a serious ecological problem on regional scales. It is believed to have caused considerable destabilization of forest ecosystems, adding to the strong structural and biogeochemical impacts resulting from exploitation history.

Deforestation and soil degradation cause the most pressing ecological problems for the time being, at least on the global scale. In many of those regions where loss of forests and soils is now high, it may be extremely difficult or impossible to restore forest ecosystems and soil productivity. Moreover, the driving forces, which are predominantly of a demographic and socioeconomic nature, do not yet seem to be lessening in strength. It can only be hoped that a wise policy of international cooperation and shared aims can cope with this problem in the future.

## INTRODUCTION

Forest ecosystems are characterized by the unique longevity of their most prominent organisms, the dominating trees. Corresponding to this longevity is a special sensitivity of the forests to long-term changes in their environment. In many of the terrestrial biomes of the earth, forests of different composition and ecophysiological characteristics form the potential natural vegetation, and they remain as greatly appreciated quasi-natural ecosystems even in the densely populated and industrialized regions of the world, where planted and managed forests have commonly replaced natural forest ecosystems.

The general environmental setting of any natural forest ecosystem uninfluenced by man would be defined by climate and soil substrate. Climate fluctuates more or less regularly and strongly on very long time scales in glaciation cycles of 100,000- to 150,000-year duration[1], as indicated in Fig. 1. Within glaciation periods and the interglacial phases as well, there occur cyclic fluctuations of temperature with a smaller amplitude and more irregular time periods in the range of 100 to 1,000 years[2], similar to the "little ice age" around the year 1600 A.D. This variability continues over annual and diurnal cycles and reaches down to the level of single weather events, which exhibit the most irregular patterns on the shortest time scales.

Soil development, on the other hand, is mostly a unidirectional, irreversible process. In all humid climates for instance, where infiltration into the soil exceeds evapotranspiration and the solum is more or less intensively leached, impoverishment and acidification of the soil will occur over extended time scales (see Matzner and Davies)[3]. It has been demonstrated that completely autochthonous forest degradations can follow such extensive soil degradation[4,5].

The picture of effects influencing forest ecosystems gets much more complex when human impact comes into play. Virtually no forest in the world today is totally uninfluenced by human activities. Even where no man ever entered a forest, it is still in contact with the atmosphere, the composition of which has already undergone global changes, the most prominent being the enrichment of $CO_2$ by about 30%. The effects of these changes on forest ecosystem dynamics are still largely unclear[6,7].

In this review, we shall focus the perspective on *managed* forest ecosystems in regions inhabited by man for a long time because they are closest to us and our experience of forests.

## MANAGEMENT OF FOREST ECOSYSTEMS: FORESTRY IN A SHIFT OF PARADIGMS

"Forestry" is not very old, in the modern sense of science-based forest management. It was established as a scientific discipline around the end of the 18th century, as a consequence of a dramatically increasing shortage of the wood resources in Europe[8,9]. Over centuries to millennia before, forest ecosystems in Europe were "managed" more or less by exploitation alone, with no regard to sustainability or conservation. A large proportion of primary forestland was cultivated, representing a tremendous land cover change happening alongside an increasing human population.

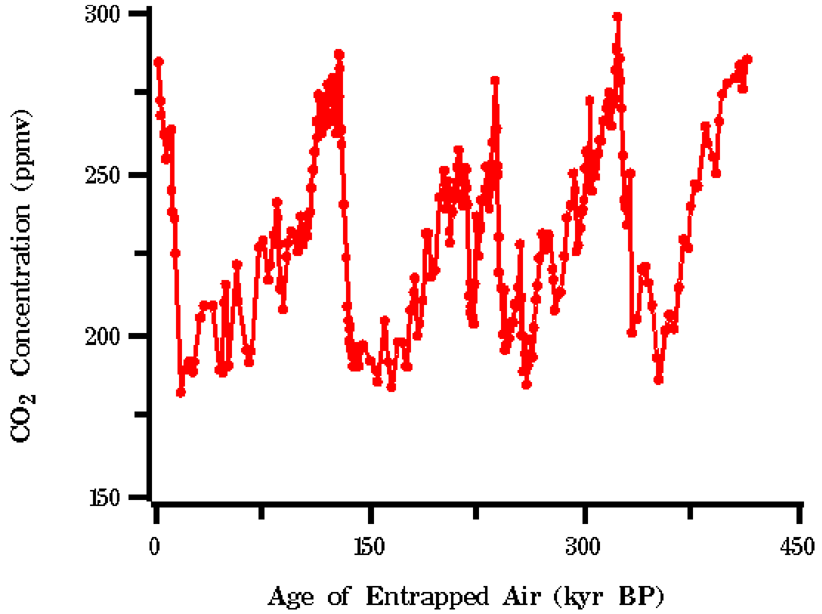

**FIGURE 1**. Long-term dynamics of atmospheric $CO_2$ concentration reconstructed from the Vostok (Antarctica) ice core. Fluctuations in global surface temperature are associated with the [CO2] fluctuations (from Barnola et al.[1]); graphics source: http://cdiac.esd.ornl.gov/trends/co2/graphics/vostok.co2.gif

Management systems developed by forest science in Europe over the past 2 centuries rely on time spans of typically 1 to 2 centuries, depending on growth characteristics of the species employed and on site conditions. In the past, the latter always have been thought to be constant over arbitrarily long time horizons. The confidence in this steadiness is expressed in the yield class tables used by foresters, as compiled for Germany, e.g., by Schober[10]. Yield class tables are empirical models based on a large number of test sites, describing the average relationship of growth and site quality, categorized in ranked "yield classes".

The soundness of established yield class tables has recently become questionable in light of substantial deviations from such models of actual tree growth in Europe[11,12]. Observations indicate that forest growth was enhanced in many locations, corresponding to a positive shift in site class of many stands. On the other hand, growth may decline where acute forest damage occurs. All these effects are probably caused by changes in site conditions over the past century due to anthropogenic influences, particularly atmospheric deposition. Atmospheric deposition has two prominent aspects: the acidification aspect and the nutrient input aspect. Although acidification of forest soils triggers several mechanisms that impair forest nutrition and ecosystem resilience[2,13,14], deposition of nutrients, particularly nitrogen, may foster tree growth[11,15]. However, potential gains in tree growth are at odds with ecological hazards arising from nitrogen eutrophication of forest ecosystems. Transfer of excess nitrogen (in the form of nitrate) to aquatic and groundwater systems is generally considered the most important risk arising from "nitrogen saturation"[16]. Additional risks identified comprise unbalanced tree nutrition and the transfer of nitrogenous greenhouse gases to the atmosphere[17,18,19].

Environmental changes other than acidification and eutrophication will also play a role in determining long-term environmental conditions of forest ecosystems, particularly the ongoing increase in atmospheric carbon dioxide concentrations, a possible climatic change with an alleged higher frequency of extreme weather conditions, and changes in silvicultural and management practices in forests.

## SCALES AND PATTERNS OF LONG-TERM INFLUENCES ON FOREST ECOSYSTEMS

This paper gives an overview of long-term environmental changes and human interferences as relevant constraints for forest ecosystems; this overview is structured by the spatial and temporal scales on which those constraints operate.

### Local to Regional Scales: Historical Land Use, Exploitation, and Degradation

Forests are the most abundant natural land cover type in the tropical, the temperate, and large parts of the boreal climate zone. Under background conditions without human interference, Central Europe, for instance, would be essentially completely covered by forest except for bogs and swamps in the lowlands and sites above timberline in the high mountains[20]. However, as part of a general feature of regions of the globe settled by humans, natural primary forests in Germany have been converted to managed production forests.

Although human land use and exploitation of natural ecosystems is a phenomenon that can be registered worldwide, its particular results show a high degree of differentiation on the local and regional scales. The reason for this is that it depends very much on the respective land use and forest exploitation systems of local and regional communities, currently and historically, and how the cultural landscapes including the remnants and transformed states of primary forest ecosystems are shaped.

Human exploitation does not only change the actual structure of forest ecosystems, but also has a lasting effect on their biogeochemical boundary conditions. In Germany and many other regions of Europe, overexploitation of forests was a common feature that often led to devastation[8,9,21]. Excessive exploitation deteriorates soil status and potential site productivity by removing nutrient capital, either directly through biomass export or indirectly by bringing about extensive mineralization and leaching losses or even soil erosion by water and/or wind. When exploitation practices lead to the mineralization and nitrification of organic nitrogen from within the ecosystem and leaching of nitrate in large amounts, this exploitation-triggered process contributes to soil acidification ($R\text{-}NH_2 + 2O_2 \rightarrow R\text{-}OH + H^+ + NO_3^-$; the proton produced acidifies the soil solid phase, whereas the mobile nitrate anion is leached from the soil with a nutrient cation equivalent).

Site quality (e.g., nutrient stores and buffer capacity) may have substantially deteriorated in the historical times of forest exploitation. With that in mind, it is hard to judge whether that exploitation together with natural disturbance events or the more recent processes of atmospheric acid deposition (discussed in the next section) have contributed more strongly to acidification and impoverishment of forest soils.

Not only has excessive harvest of timber led to overexploitation in past centuries, but other forms of forest utilization, which exerted additional and sometimes even stronger effects, also had detrimental consequences on site quality. Such additional exploitations effectuated further exports of nutrients, often exceeding the export by timber utilization[22], and caused obstruction of natural regeneration by domestic animal feeding (e.g., cattle, sheep, and goats feeding on saplings and young trees as well as pigs feeding on acorns), and deterioration (compaction by trampling and erosion) of soil by cattle fed in the forest. Most prominent among the historical exploitation practices was litter raking, i.e., the export of leaf litter and surface humus from the forest for use in farm stables. Forest litter was brought into stables as a substitute for straw, was then mixed with and enriched by animal manure, and then plowed into the fields. Hence, the whole process was an export of nutrients and humus (the latter being important for soil structure) from forests to arable land. This led to an improvement of fertility in arable soils, which could only be

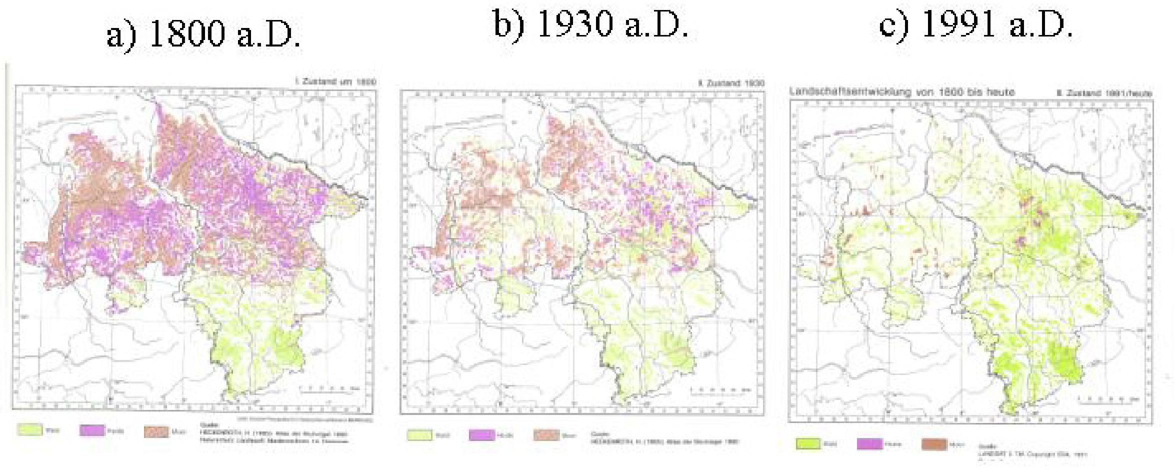

**FIGURE 2**. Historical change in land use and forest cover within the past 2 centuries in Lower Saxony, northern Germany[23]; green = forest, violet = heathland, brown = peat bogs.

achieved in this way, since modern synthetic fertilizers were not available. Even the access to natural materials for soil improvement (like limestone) was limited in areas where such resources were too far away. The moderate gains in fertility of field soils, however, were paid for by considerable impoverishment of forest sites.

Almost throughout Europe, the forests were also used as a ground for cattle, goats, and pigs. This practice led to disturbances of natural regeneration. Moreover, it is likely that erosion by water occurred on steeper slopes, where cattle disturbed the surface soil. Twigs and leaves were also taken from living and harvested trees and used as fodder in times when other fodder sources were scarce. This form of exploitation not only contributed to nutrient export from the forests, but also to a further deterioration of forest structure.

The historical overexploitation of forests gravely impaired site quality. Typical final states of the forest after long-term use of these practices were open, degraded forest landscapes with only a few old trees remaining, or expanded heathlands with a dwarf shrub cover on highly impoverished soil. (Many old paintings from the medieval time up to the 18th century show graphically such states of the landscape.)

A cartographic representation of the tremendous change in land use and land cover over the past 200 years is given in Fig. 2 for the area of Lower Saxony, Germany[23]. In 1800, the northern lowlands were either covered with primary peat bogs or with heathlands as degradational states of former forest (or abandoned arable) land (Fig. 2a). In the mountainous areas of the southernmost part, forests existed largely in heavily exploited states[9], and with a smaller total area than today. Although Fig. 2a marks in time the approximate onset of sustainable, science-based forestry, Fig. 2b (at 1930 A.D.) depicts the ongoing processes of replanting forests in degraded heathland areas and reclaiming land for cultivation from the peat bogs. (Note that a tremendous shift in carbon sequestration status of the soils in the region must have gone along with the conversion of the bogs, since the peat was also being burned for heating and cooking.) Fig. 2c shows the approximate present status, with forests having regained a larger part of the land area and having a much higher biomass stock than formerly.

Thus, while site quality and forest ecosystem structure suffered impairment over centuries of exploitation, the modern European land-use pattern and, in fact, modern civilization was formed during that time. Forest management was generally not sustainable; rural and urban ecosystems were developed and sustained at the expense of forest ecosystems. Although the overall time scale of nonsustainable forest exploitation in Europe extends over more than a millennium, spatial scales of interference are smaller and of a more individual nature, depending on local or regional traditions and preferences in land use. Therefore, it is eminently important to consider site history in appraising actual ecosystem status, site quality, and potential in forest management.

## Regional to Continental Scales: Drift of Chemical Soil Status in Forests Caused by Long-Range Air Pollution

In the industrialization period, a more recent chapter of history, new pressures on forest ecosystems and their soils occurred. Air pollution adjacent to emission sources often reached such levels that direct damage to forest vegetation occurred. This phenomenon was described as "smoke damages" (*Rauchschäden* in German) and was reported early in the literature, e.g., Schroeder and Reuss[24] and Wieling[25].

In addition to such strong direct effects under high air pollutant concentrations, another process developed that was slower but spatially more widespread. Components of air pollution, chemically transformed into strong mineral acids ($H_2SO_4$, $HNO_3$, HCl), advanced soil acidification further than already attained by exploitation practices. Unlike the soil acidification process by excessive organic matter export, acid deposition introduced large amounts of mobile, conservative anions (predominantly sulfate) into the soil water, thus facilitating aquifer- and surface-water acidification. It is probable that this aspect of anthropogenic atmospheric acid deposition is even more important ecologically than the further contribution of acid rain to forest soil solid phase acidification. Detailed reviews of the chemical processes involved in soil and water acidification can be found[13,26,27,28,29].

It is noteworthy that the period of industrialization and augmentation of acid deposition coincided approximately with the development of scientifically based modern forestry, which put an end to overexploitation and was successful in recuperating and sustaining harvests of timber from forests. With the end of excessive utilization practices, a prospect of recovery was given for forest soils, at least to some extent.

So it would seem there were two opposing developments operating at the same time: (1) on the one hand, recovery of forest structure and of soil organic matter, offering some potential for a slow recovery of soil structure and of nutrient stores; and (2) on the other hand, acid deposition that played an important role in deteriorating site quality, as was directly evidenced in some comparisons of chemical parameters in forest soils over long time spans[30,31]. The present extent of forest soil acidification in Germany is illustrated in BMELF[32].

It is generally supposed that acidification led to regional- to continental-scale destabilization of forest ecosystems in intensely industrialized regions (e.g., Central Europe, the Eastern U.S., Japan). A "novel type forest decline" (*neuartige Waldschäden* in German), caused by multifaceted stress mechanisms related to acidification, was discerned[33,34].

Changes in rooting patterns as a consequence of acidified soil have been demonstrated in several case studies[35,36]. Long-term monitoring in some case studies, e.g., the German Solling Project, revealed a turn of the trend in sulfur and acidity deposition beginning at the end of the 1980s[37]. The reasons for this change in trend were air pollution legislation in West Germany and the closing of highly polluting industries in the eastern part of Germany and former Czechoslovakia[38]. The alleviation of atmospheric acidity loads to forest soils offered an opportunity to stabilize element cycles in forest ecosystems. The potential for a relatively fast recovery of *soil water* chemistry was demonstrated in an ecosystem manipulation experiment with application of artificially prepared, "preindustrial" precipitation[39,40]. However, the impaired rooting patterns and the strongly acidified *soil solid phases* (often with large amounts of acidity stored in labile or meta-stable binding forms) represent an ecological long-term memory of acidification that can only be gradually annulled[32,35].

Root systems apparently adapt to worsening soil chemical conditions by a loss of fine root biomass, a dieback of the root system in the deeper mineral soil, and a shallow rooting pattern, restricted to upper soil layers that are influenced by humic compounds that are detoxifying monomeric aluminum ions. Together with a distinct aluminum enrichment in deeper roots, these findings can be interpreted as evidence in favor of the aluminum toxicity hypothesis. However, experimental results concerning aluminum toxicity under field conditions are somewhat ambiguous[41,42].

In addition to soil acidity, another issue of concern with respect to long-term effects is high atmospheric nitrogen deposition into forest ecosystems. In contrast to sulfur and acidity inputs, no decreasing trends can be discerned clearly for nitrogen compounds[43,44]. Nitrogen inputs from the atmosphere can have a fertilizing effect and may foster tree growth[15], but they may also lead to nutritional imbalances, giving rise to stress effects[34,45,46]. A further, significant potential of ecological risk emanates when excess nitrogen is converted to nitrate in nitrogen-saturated forest ecosystems, which can be menacing to aquatic ecosystem and water resources, or when nitrogen is converted to greenhouse gases and emitted from forest soils[17,34,47,48].

These interactions and trends persist together with an unbroken trend of rising $CO_2$-concentrations in the atmosphere and the possibility of climatic changes. They are forming a constellation of rapid and complex environmental modifications to which the forests in industrialized regions were not exposed during their evolution, and the consequence of which cannot be predicted with certitude.

The patterns and processes discussed above refer preferentially to industrialized regions in developed countries or those strongly developing at present, e.g., India and China. They operate rather uniformly at spatial scales larger than those relevant for the historical land-use practices considered in the last section (i.e., regions to parts of continents). Temporal scales range from decades to almost 2 centuries, depending on the duration of industrialization.

## Global Scale: Deforestation and Soil Degradation

Loss of forests and the concurrent degradation of soils is a pressing ecological problem affecting large areas of the globe. It is subject to the particular climatic, pedological, topographical, and socioeconomic settings in the respective regions; hence it appears in various forms.

Soil degradation may be induced by human interference or come about as a natural process. Numerous examples of anthropogenic degradational processes are given in WGBU[49]; a pattern of autochthonous, wholly natural soil and forest degradation dynamics was reported by Fölster[4,5]. However in the vast majority of cases, soil degradation seems to be related to past and present human activities[50], particularly those connected to land use, and above all, forest clearing with the subsequent cultivation and exploitation of land.

The problem is not exclusively modern, as a famous quote of the Greek philosopher Plato (from his writing *Kritias*) may illustrate: "As a result (of overgrazing) Attica had become deforested, the soils depleted, and there are remaining only the bones of the wasted body – all the richer and softer parts of the soil having fallen away." [1]

[1] There has been considerable debate about the appropriate understanding of this quote in environmental science and history. Many authors claim that the Mediterranean is an excellent example for long-term sustainability of a cultural landscape; for more details on this discussion see [21], p. 160 ff.

**TABLE 1**
**Land Area and Land Use**

| | Land Area | Arable Land Incl. Cropland [million ha] | Pasture and Grasslands | Forest and Woodland |
|---|---|---|---|---|
| Africa | 2,964 | 188 | 853 | 761 |
| North and Central America | 2,178 | 271 | 362 | 855 |
| South America | 1,753 | 103 | 495 | 846 |
| Asia | 2,679 | 469 | 800 | 535 |
| Europe | 473 | 136 | 80 | 158 |
| Former USSR | 2,190 | 229 | 344 | 823 |
| Oceania | 845 | 51 | 428 | 200 |
| World | 13,082 | 1447 | 3,362 | 4,178 |

From Ref.[51]

Roughly 9 billion ha — or 69% of the 13 billion ha of total land surface — are vegetated. Natural and seminatural ecosystems in the form of forests, woodlands, and grasslands under different intensities of human utilization and management still account for the largest part, while ca. 1.4 billion ha or 11% are currently estimated to be intensively managed arable land (see Table 1)[51].

It is estimated that almost 2 billion ha of land have undergone degradation processes (22% of the vegetated land area), the most important ones being erosion by water and wind (ca. 1.65 billion ha) (see Table 2). Regions most severely affected by soil degradation are frequently those with a high human population density or with a strong technical human impact (e.g., highly mechanized large-scale farming in the "dust bowl" of the U.S. Midwest), or semiarid regions with marginal productivity.

Besides erosion processes, chemical and physical alterations may lead to degradation. Although the area affected by the latter is much smaller than that affected by water and wind erosion (see Table 2), they are almost exclusively caused by human interference. In the preceding section, large-scale acidification of forest soils in Central Europe by long-range air pollution and acid deposition was discussed as an example.

**TABLE 2**
**Types of Soil Degradation and Areas Affected [million ha][50]**

| Types of Degradation | Light | Moderate | Strong/Extreme | Total |
|---|---|---|---|---|
| Water erosion | 343 | 527 | 224 | 1,094 |
| Wind erosion | 269 | 254 | 26 | 549 |
| Chemical degradation | 93 | 103 | 43 | 239 |
| - loss of nutrients | 52 | 63 | 20 | 135 |
| - salinization | 35 | 20 | 21 | 76 |
| - contamination | 4 | 17 | 1 | 22 |
| - acidification | 2 | 3 | 1 | 6 |
| Physical degradation | 44 | 27 | 12 | 83 |
| Total | 749 | 911 | 305 | 1,965 |

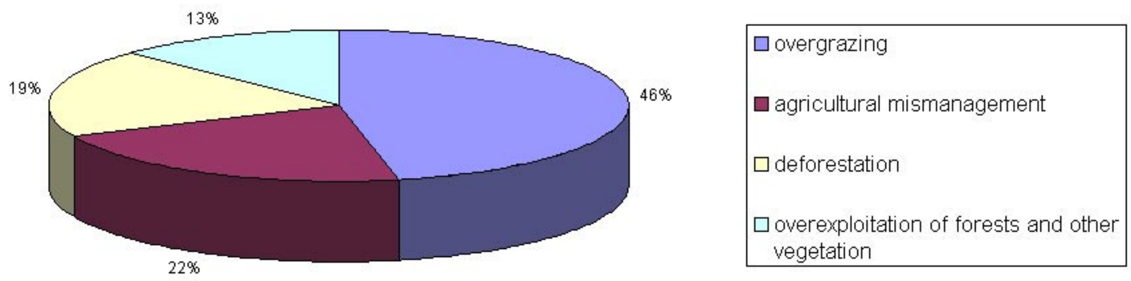

**FIGURE 3**. Causes of global land degradation[54].

The major causes of soil degradation worldwide are deforestation, overgrazing, agricultural mismanagement, excessive export of soil nutrients in harvested crop and wood biomass, sealing of soils for urban and traffic constructions, local contamination by disposal of (toxic) wastes or large-scale pollution by atmospheric deposition, and irrigation of arid land in an unsustainable manner, bringing about salinization of the soil. Also, the loss of organic matter in soils due to inappropriate management practices can be considered as a kind of chemical degradation.

Most of the assessments of current global loss of forests give a rate of ca. 12 million ha/year (e.g., http://www.bundestag.de/ftp/pdf_arch/au_14_29.pdf), an area larger than the entire forest land of Germany (ca. 10 Mha). In spite of an increased public awareness for the protection of forests, the rate of forest loss has not slowed in the recent past. It is particularly severe in the developing countries, where 180 million ha of forest were cleared between 1980 and 1995 according to the FAO; this figure is a net balance, i.e., it includes afforested areas. In some individual regions in developing countries, enormous rates of deforestation have been evidenced. For instance, a study from South Sumatra, Indonesia, reports a regional decline in primary forest area from 57% in 1970 13% in 1990[52]. The pressure on the forest in this case resulted from transmigration and the resultant effect of an increased population. Even without substantial population growth, changes in the socioeconomic and political environment faced by shifting cultivators can bring about dramatically increased rates of primary forest conversion, as was shown by Lawrence et al.[53].

In contrast to that, the forested area in Europe increased by ca. 4% between 1980 and 1994. This pattern clearly indicates the role of socioeconomic pressures in deforestation and will be further discussed below.

In the literature, the part of deforestation in overall global soil degradation is placed mostly in the following ranking: overgrazing > agricultural mismanagement > deforestation > overexploitation of forests and other vegetation (Fig. 3)[54]. Yet, in many situations it is evident that forest clearing precedes (or preceeded) the other causes of soil degradation listed above since they often come into effect on land that was formerly cleared of its forest cover.

Finally, it should be mentioned that the data basis underlying global soil degradation appraisals is somewhat fuzzy, as is reflected in considerable deviations between different assessments performed in the recent past (see Fig. 4)[54]. The total amount of degradation estimated differs between those studies just as much as the distribution between classes of severity. This is owing to the fact that there is currently no soil degradation database that rests upon rigorous quantitative data, but very different methodologies were employed, e.g., questioning of regional experts in the GLASOD study vs. statistical information from governments in FAO/UNEP. Moreover, some authors point to the fact that humans have also made soils more fertile on a large scale, providing improved food resources and security[55]. However, in spite of the uncertainty and complexity involved, all assessments arrive at hazardous figures and ratings of actual global soil degradation, which is strongly and intimately linked with a global retreat and conversion of forest ecosystems.

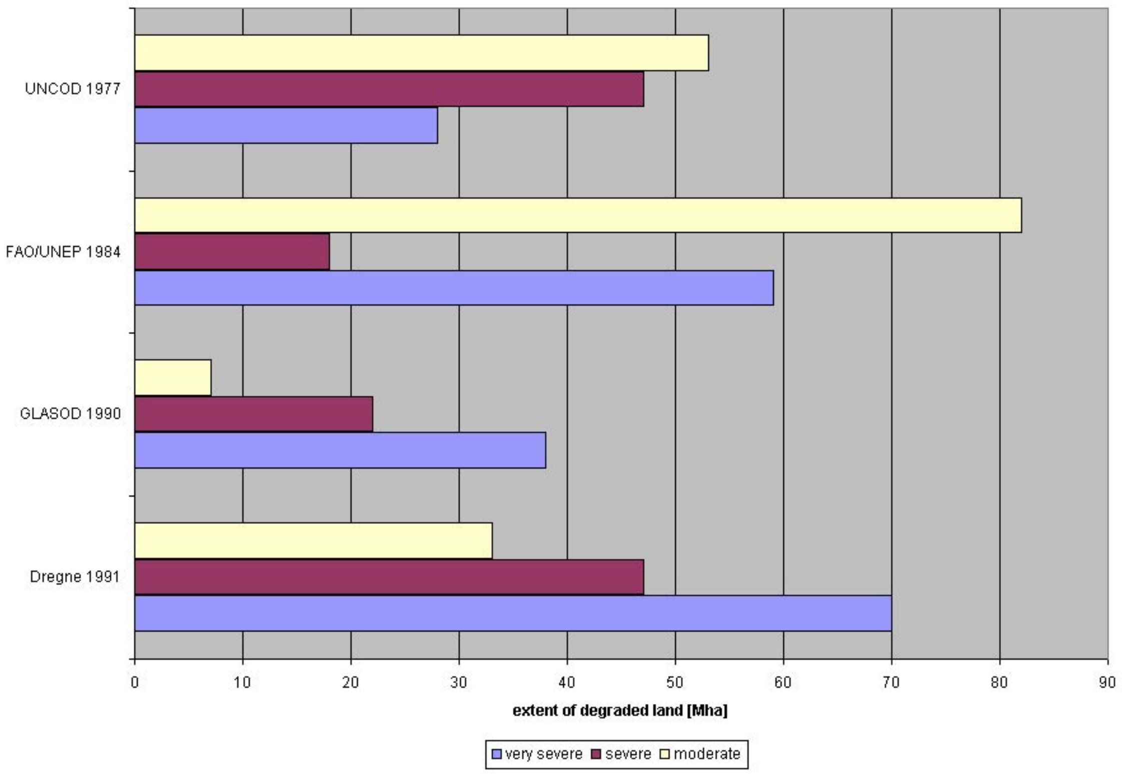

**FIGURE 4**. Different assessments of global soil degradation published[54].

## DISCUSSION: APPRAISAL OF LONG-TERM EFFECTS ON FORESTS

The description of long-term effects on forest ecosystems given above included only effects already observed in the past and their scales in space and time. A topic of great interest in ecological research at present is the possible global climatic change as a consequence of anthropogenic greenhouse gas emissions. It is fairly uncertain and under debate within the scientific community how strong an upcoming climate change would be and what would change in particular, over which times scales it would develop, and what exactly would be the consequences for forest ecosystems worldwide. Among suspected effects, there can be most prominently found in the literature:

- Changes in microbial decomposition of soil organic matter, with both suspected positive and negative feedbacks on atmospheric $CO_2$[56,57]; however, there are also studies in which no effects could be observed[59].
- Forest growth responses to elevated atmospheric $CO_2$; most authors presume positive growth response and hence a negative feedback effect on atmospheric $CO_2$, which would, however, be quite limited[6,7,59,60].
- A potential to sequester more carbon in forest soils by nitrogen addition (N fertilization) or due to atmospheric deposition of nitrogen[61,62,63] and due to adapted forest management strategies[64].
- Increased occurrence of forest fires in regions where droughts occur more frequently and heavily as a consequence of climate change. Frequent large-scale forest fires would most probably be a strong positive feedback on atmospheric $CO_2$. However, there is plenty of evidence in the literature that fire is not always and not only ecologically detrimental, but belongs to the normal successional cycle in many types of forest ecosystems[65,66,67,68].

Other suspected effects of global climate change have been brought up, which are discussed in more voluminous reviews[49,69], and still many more may emanate as the scientific discussion continues. A comprehensive review with respect to impacts on forest ecosystems appeared most recently[2].

For the time being, the problem to rate possible effects is the uncertainty inherent in climate change predictions. It is probably as impossible for us now to make reliable predictions on a time horizon of 100 to 200 years, as it would have been for Humboldt, Darwin, or Goethe to predict our present world of airplanes, telephones, and computers. Under these conditions, possible climate change effects shall not be included in the endeavor of grading long-term effects on forest ecosystems performed below. However, if strong and rapid climatic changes should occur, there could be tremendous effects on the forests, since climate is doubtlessly the strongest environmental force governing their existence. The particular kind and magnitude of effects would again strongly depend on the region of the globe considered.

Among the other long-term effects discussed in the Global Scale section, the exploitation history of forests presumably had the biggest effect on their present structure and function. This appraisal relies on the fact that human utilization of forests in regions with a long history of settlement has completely changed and formed the forest ecosystems over very long time spans. In some regions the process ended in widespread degradational states of former forest ecosystems like shrublands in the Mediterranean or extensive grass- and heathlands as in Britain. In others, biomass-rich and productive forests were re-established, which differ, however, from the original forest ecosystems in many structural and probably also some functional features. It is important to note that re-establishment of forests after long-term devastation is much more favored in humid environments with younger soils (like northern and central Europe) than it would be in semiarid or pedologically old tropical environments.

Soil acidification by atmospheric acid deposition is a problem that only affects forest ecosystems in some regions of the globe, namely those in humid climates and under high atmospheric acid input. In those regions, particularly Central Europe, it significantly adds to soil acidification brought about by the humid climate, natural disturbances, and human exploitation history. It is difficult to judge if exploitation history or acid deposition have contributed more to the current acidity status of forest soils in those areas; the appropriate result of such an appraisal may vary regionally or even locally. In the scientific discussion on the significance of "acid rain" that took place during the past decades, it may have been overlooked to some extent that natural disturbance events like windthrow can also lead to strong mineral acid release in forest soils (i.e., nitric acid production from soil organic N). Such disturbances, as the "Lothar" storms in France and Germany on Christmas 1999, are pretty rare on our human time scales of perception, but considering the very long time spans relevant for forest soil development, they appear like a regularly recurring phenomenon. However, those effects are not quantified and cannot be quantified *a posteriori*. Beyond question, acid deposition has exerted strong effects on the forest soils of Central Europe, and even stronger effects on surface water chemistry, owing to the huge quantities of mobile anions introduced by this anthropogenic environmental change.

The human influences illustrated in the last part of the Global Scale section, deforestation and soil degradation, cause the most pressing ecological problems for the time being, at least on a global scale. In those regions where loss of forests and soils is now high, it may be difficult or impossible to restore ecosystems and soil productivity. Moreover, the driving forces, which are predominantly of a demographic and socioeconomic nature, do not yet seem to be lessening in strength. It can only be hoped that a wise policy of international cooperation and shared aims can cope with this problem in the future.

## CONCLUSION

In this brief review, long-term effects on forest ecosystems under direct human impact were analyzed for their consequences with respect do different scales in space and time. Three domains of long-term influences were identified:

- Exploitation and conversion history of forests in areas of extended human settlement
- Long-range air pollution and acid deposition in industrialized regions
- Current global loss of forests and soil degradation

The third point in the list above corresponds strongly with the first one. Cultivation — with its tremendous land cover changes — took place in Europe many centuries ago and expanded over centuries. Although deforestation is a phenomenon predominantly observed in the developing countries, it threatens biotic and soil resources on a global scale. It may be impossible to motivate people to perform forest conservation while they must gain their livelihood from cultivating forestland. Moreover, it would be inadequate from an ethical point of view, since the developed countries have performed exactly such practices in their history. The process there ended in today's "cultural landscapes", which are for the most part highly valued by their inhabitants and stakeholders. It can only be hoped that in the developing countries a similar equilibrium between land use and land resources can be attained, in which an adequate proportion of forest can be conserved. It is probably an illusion, however, to expect pressure on the forests to lessen as long as the major driving forces, i.e., population growth and the demand for more land for food production, are in operation.

It is hoped a solution to this challenge can be found in a medium-term time horizon. One way to take pressure off the forest would certainly be to enhance productivity of existing cultivated land and provide people with income from that productivity. In the developing countries, however, this is still subject to economic constraints (availability of money for infrastructure, machinery, fertilizers, etc.).

If an equilibrium of population and land resource can be reached, it will be very important to manage the forests in a manner suitable to the needs of people, while at the same time securing structures and functions of the managed forest ecosystems. A completely conservational approach cannot be successfully pursued in populated regions where people rely on income and other benefits from the forest (e.g., recreation). However, there should be room for some natural forest reserves to conserve and protect as much biodiversity possible, even in densely settled environments. Expanded natural forest reserves will persist without a problem in areas where human access and interference are small.

Consequently, in a realistic approach to secure our forest heritage in the long-term perspective, it should be regarded as equally important to manage and utilize our forests in the populous regions, thus securing their functions for nature and human society, as it is to protect and conserve natural ecosystems in more remote areas.

## ACKNOWLEDGMENTS

I wish to express my gratitude to Prof. Dr. Dr. h.c. mult. Bernhard Ulrich, my academic teacher for over 15 years, for all I could learn from him and for the encouragement he gave to his scholars to find their own scientific point of view.

## BIOSKETCH

**Michael Bredemeier** is a Senior Scientist in Research and Teaching at the Forest Ecosystems Research Center of Goettingen University, Germany. He holds a Ph.D. in Soil Science (1987) and Habilitation in Soil Science and Forest Nutrition (1999). His research interests include terrestrial ecosystems ecology, forest ecology, soil science, soil chemistry, element cycling and budget studies, ecological time series analysis, and long-term dynamics in ecosystems.
